# An Extract Purified from the Mycelium of a Tomato Wilt-Controlling Strain of *Fusarium sambucinum* Can Protect Wheat against Fusarium and Common Root Rots

**DOI:** 10.3390/pathogens7030061

**Published:** 2018-07-14

**Authors:** Larisa A. Shcherbakova, Tatyana A. Nazarova, Oleg D. Mikityuk, Ekaterina A. Istomina, Tatyana I. Odintsova

**Affiliations:** 1 Laboratory of Physiological Plant Pathology, All-Russian Research Institute of Phytopathology, B. Vyazyomy, Moscow Reg.143050, Russia; borisgurevich@list.ru (T.A.N.); mod-39@list.ru (O.D.M.); 2 Laboratory of Molecular-Genetic Bases of Plant Immunity, Vavilov Institute of General Genetics, Gubkina str. 3, 119333 Moscow, Russia; mer06@yandex.ru (E.A.I.); odintsova2005@rambler.ru (T.I.O.)

**Keywords:** wheat root rots, biocontrol, fungal extracts, induced resistance, defensins, field trials

## Abstract

An approach to manage seed-transmitted Fusarium crown-foot-root rot (FCR, *Fusarium* spp.) and common root rot (CRR, *Bipolaris sorokiniana*) on wheat, avoiding environmental risks of chemicals, is seed treatments with microbial metabolites. *F. sambucinum* strain FS-94 that induces resistance to tomato wilt was shown by this study to be a source of non-fungitoxic wheat-protecting metabolites, which were contained in a mycelium extract purified by gel-chromatography and ultrafiltration. Plant-protecting effect of the purified mycelial extract (PME) was demonstrated in vegetation experiments using a rolled-towel assay and by small-plot field trials. To elucidate mechanisms putatively underlying PME protective activity, tests with cultured *Triticum aestivum* and *T. kiharae* cells, particularly the extracellular alkalinization assay, as well as gene expression analysis in germinated wheat seeds were used. Pre-inoculation treatments of seeds with PME significantly decreased the incidence (from 30 to 40%) and severity (from 37 to 50%) of root rots on seedlings without any inhibition of the seed germination and potentiation of deoxynivalenol (DON), DON monoacetylated derivatives and zearalenon production in FCR agents. In vegetation experiments, reductions in the DON production were observed with doses of 0.5 and 1 mg/mL of PME. Pre-sowing PME application on seeds of two spring wheat cultivars naturally infected with FCR and CRR provided the mitigation of both diseases under field conditions during four growing seasons (2013–2016). PME-induced ion exchange response in cultured wheat cells, their increased survivability, and up-regulated expression of some defensins’ genes in PME-exposed seedlings allow the suggestion of the plant-mediated character of disease-controlling effect observed in field.

## 1. Introduction

Root rots caused by soil fungi result in yield losses of various cereals including wheat, which is a crop of great economic importance for many grain-producing regions worldwide [1,2,3,4,5,6,7,8]. In Russia, Fusarium crown-foot-root rot (FCR) as well as common root rot (CRR) are damaging diseases wherever wheat and barley are grown, but generally FCR is of more concern because of fusariotoxins and association with scab of cereals. Although FCR caused by several *Fusarium* spp. is more typical for cooler wheat-growing areas, while CRR caused by *Bipolaris sorokiniana* (syn. *Helminthosporium sativum*, teleomorph: *Cochliobolus sativus*) prevails in southern regions, these fungi often occur together, forming the Fusarium-Helminthosporium root rot pathogenic complex in the spring wheat rhizosphere, where the composition and proportion of the rot-inducing species vary depending on local climate, soil type and cropping systems [3,9].

Since FCR and CRR belong to seed-transmitted diseases, infection of seed significantly contributes to pre- or post-emergence death of young seedlings [9,10,11,12,13,14,15]. The risk of plant rotting increases after sowing under unfavorable conditions when seeds sit long in the cool or wet heavy soil, hence pre-sowing seed treatment with fungicides is the primary tool for reduction of seedling damage [16,17,18,19,20]. Along with fungicides, crop rotation, antagonistic or growth promotion bacteria and fungi [5,21,22,23,24,25], as well as their metabolites with fungicidal or resistance-eliciting activity, and chemicals mimicking biogenic elicitors [6,26,27] are used for wheat seed treatment to reduce FCR and CRR. However, chemical pesticides are not always equally effective against *B. sorokiniana* and all *Fusarium* species. Moreover, fungicidal treatments sometimes stimulate mycotoxin production in Fusaria [13,28], and herbicide application can lead to the increased colonization of wheat and barley plants or crop residues by these fungi [29]. Crop rotation alone can be ineffective or even increase the risk of FCR and Fusarium head blight development [30]. Seed treatments with biocontrol microorganisms or their biomolecules conjointly with other management procedures (healthy seeds, resistant cultivars, rotation with appropriate non-host crops, correct selection of fertilizers) might be an approach for improving the systems of integrated wheat protection from FCR and CRR without increasing the chemical impact in cereal-growing regions [19]. To implement this approach, biocontrol microorganisms, potential biopesticides or resistance inducers, which would effectively reduce the diseases on wheat plants after application on seeds should be available [31,32]. Several years ago, we discovered that *F. sambucinum* FS-94 induced systemic resistance to the vascular wilt of tomato [33], and briefly reported the inhibition of some rot- or blotch-causing wheat pathogens by the metabolites of this fungus under controlled and field conditions [34,35]. Continuing these investigations, in the current work we examine the effect of FS-94 metabolites on FCR and CRR agents more entirely, analyze previous studies and report new findings of laboratory and field experiments on wheat protection using the seed treatments with the extract obtained from FS-94 mycelium.

## 2. Results

### 2.1. Protection of Wheat Seedlings with the Purified Mycelial Extract (PME) against Root Rot Pathogens in Vegetation Experiments with Artificial Seed Inoculation

**Figure 1:**
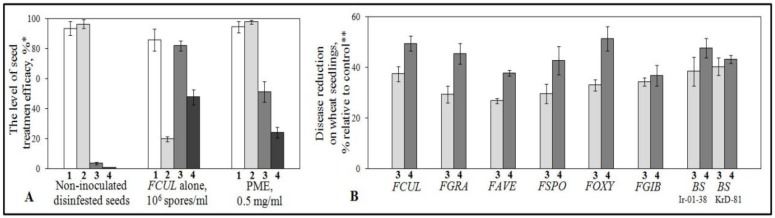
Histograms showing effects of Enita seed treatments with purified extracts of *F. sambucinum* mycelium (PME) prior to artificial seed inoculation with *F. culmorum* (**A**), other pathogenic Fusaria and two strains of *B. sorokiniana* (**B**) on seed germination and the pathogen development on seedlings. **1**- Seed germination, %; **2**- Number of seedlings emerged from germinated seeds, %; **3**- Number of symptomatic seedlings, %; **4**- Disease severity (DI%). *FUCL*- *F. culmorum*, *FGRA*- *F. graminearum*, *FAVE*- *F. avenaceum*, *FSPO*- *F. sporotrichioides*, *FOXY*- *F.oxysporum*, *FGIB*- *F. gibbosum*, *BS*- *B.sorokiniana*. *Average from three experiments, 200 wheat kernels per treatment in each. Bars in the histograms represent standard error of mean (SE). **Control - The levels of FCR or CCR development on seedlings grown from seeds treated with sterile distilled water and inoculated by the corresponding pathogens (1–1.5 × 10^6^ conidia/mL).

Initially, aquatic solutions of freeze-dried PME at three concentrations (0.005, 0.05 or 0.5 mg/mL) were used for seed soaking before their inoculation with the suspension of *F. culmorum* conidia to estimate seedling damage by a rolled-towel assay. The lowest concentration applied was inactive, seed exposure to the middle one (0.05 mg/mL) resulted in a statistically insignificant reduction of the rot severity (by 1.2%) and did not decrease the disease incidence compared to control, while PME at a concentration of 0.5 mg/mL significantly increased the number of seedlings emerged from inoculated seeds compared to non-treated inoculated control, as well as decreased both the number of symptomatic seedlings and the disease index without any inhibition of seed germination (Figure 1A). Moreover, vegetation experiments involving pre-inoculation seed treatments with PME at 0.5 mg/mL revealed an evident protection against all tested pathogenic strains of *Fusarium* spp. and *B. sorokiniana*, which was accompanied by 30–40% reduction in the incidence and from 37 to 50% mitigation in severity of root rots (Figure 1B). Further increase in PME concentration to 1.0 mg/mL resulted in no key enhancement of antipathogenic effect.

The minimal percentages characterizing additive protection effect (sum of reductions in the incidence and severity mitigations of each pathogenic strain) were found when seeds treated with PME at a concentration of 0.5 mg/mL were inoculated with *F. avenaceum* (about 65%), while the maximal additive effect was observed in case of inoculations of PME-treaded seed with *F. culmorum* (86.8%), *F. oxysporum* (84.2%) and *B. sorokiniana* (86.1% and 86.2% for Ir-01-38 and KrD-81, respectively. Since filtrate of culture liquid (CL) has been reported previously to inhibit spore germination of some plant pathogenic fungi [36,37], and protected wheat plants in field from *Stagonospora nodorum*, another wheat pathogen affecting seeds [35], CL ability to protect wheat against FCR and CCR agents was tested in this research. In our experiments, FCL did not prevent the development of *Fusarium* spp. on the seedlings, possessed a low suppression activity toward the CRR agent, did not inhibit germination of conidia in these fungi (data not shown), and was excluded from the further studies.

### 2.2. Plant-Mediated Character of the Protective Activity

**Figure 2:**
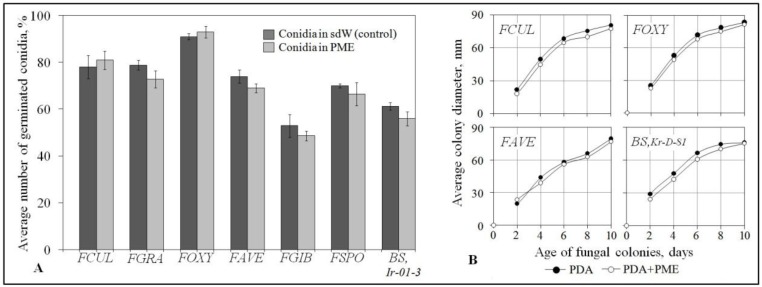
Histogram and curves demonstrating the lack of toxic impact of *F. sambucinum* mycelium extract (PME, 0.5 mg/mL) toward FCR and CCR agents. *FUCL*, *FGRA*, *FAVE*, *FSPO*, *FOXY*, *FGIB*, *BS* - see Figure 1. Mean values of three independent experiments comprising in vitro germination of conidia (**A**) and radial growth (**B**) assays (300 conidia and five colonies of each pathogen per a treatment in each of them) are presented. Bars in the histogram (**A**) represent SE; sdW- sterile distilled water. The representative data of radial growth test (**B**) are shown for four pathogens as the growth dynamics of all eight pathogenic strains was found to be similar, and no inhibitory effects were also revealed with PME at 1.0 mg/mL. Differences of means among sizes of PME-exposed and control colonies grown on potato dextrose agar (PDA) are insignificant (*p* > 0.05, bars are not shown) in all cases.

No inhibition of conidium germination or retardation of the colony growth was found when all stains tested of the causative agents were exposed to PME at the pant protecting concentration (Figure 2). These data pointed to FCR and CCR suppression by the extract was not associated with its toxicity for the pathogens but could be plant-mediated.

**Figure 3:**
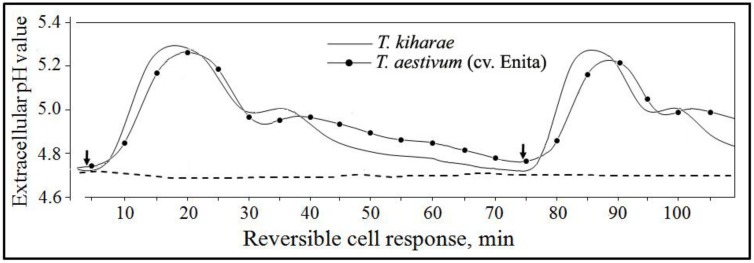
Typical profiles of a reversible change of extracellular pH in cell suspensions of two wheat species in response to addition of FS-94 mycelium extract (PME) that reduce FCR and CRR incidence and severity on artificially infected wheat seedlings. The representative data are out of one of three experiments with PME samples independently isolated from FS-94 mycelium. **3**- Dashed line shows extracellular pH of non-treated cells. Arrows indicate starting points of cell treatment and re-treatment with PME.

Indeed, PME induced the ion exchange response in wheat cells, one of early plant defense reactions involved in plant-pathogen interactions and augmented the cell tolerance to lytic activity of *F. culmorum*. Thus, addition of the lyophilized PME preparation to a final concentration of 0.5 mg/mL led to a reversible change of extracellular pH in cultured cells of *Triticum aestivum* (cv. Enita) and *T. kiharae* with a nearly similar response profile and close Δ pH values. Responsive alkalinization of the extracellular medium started to increase after a 5 min lag phase and reached a maximum to 15 min (*T. kiharae*) or 20 min (cv. Enita) followed by a gradual subsidence over the next 60–70 min (Figure 3). A reversible pH change occurred in response to PME re-addition, thereby showing that cells saved their viability and responsiveness after the treatments.

Pre-incubation of wheat cells with the tested extract was also found to promote their survival upon subsequent pathogen inoculation. Almost all Enita cells were lyzed by germinated fungal conidia in the non-treated suspensions compared to control ones after a 48-hour co-cultivation, while at least a half the PME-treated cells survived at the presence of conidia during the same period (Table 1).

An increased survivability was also observed in cultured cells of a highly resistant wheat *T. kiharae* exposed to PME before they contacted with the pathogen. In this species, treatment with PME resulted in 89.8–94.0% cell survival on the second co-culturing day, and the number of unlzyed cells in the PME-exposed suspension (28.3 ± 0.92 × 10^3^) was four time higher compared to non-exposed inoculated control (7.1 ± 1.39 × 10^3^) by the end of the co-cultivation.

Experiments with germinated seeds confirmed plant-mediated type of the protection and showed treatment with PME is accompanied by expression of plant defensin genes.

**Figure 4:**
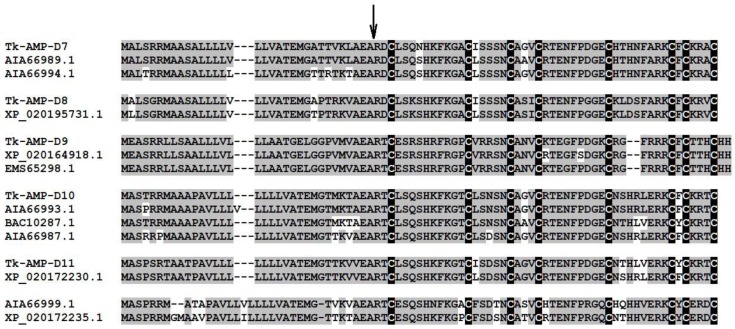
Amino acid sequence alignment of *T. kiharae*, *T. aestivum*, and *Ae. tauschii* defensins. Identical residues are shadowed gray. The position of the mature peptide is shown by an arrow. Cysteine residues are highlighted white on the black background.

To demonstrate that, we first screened the deposited in GenBank sequences using the BLAST 2.0 algorithm with *T. kiharae* Tk-AMP-D2 defensin sequence as a query. Ten defensin-like sequences from *T. aestivum* and its closest relative *Aegilops tauschii* were selected, and their mRNA sequences were retrieved from the National Center for Biotechnology Information (NCBI) database. *T. kiharae* defensin-like genes were isolated from the cDNA of embryonic shoots with emerged leaves by PCR amplification with gene-specific primers (see Table 5), which were designed to the ends of the protein-coding regions of these genes. PCR products of the necessary size were eluted from the gel, cloned, and sequenced. Of 10 selected defensin-like genes, primers to 5 genes produced amplification products with the cDNA from PME-treated *T. kiharae* samples as compared to control. Amino acid sequence alignment of the deduced amino acid sequences of defensin precursors is shown in Figure 4.

Five defensin-like genes were thus discovered in *T. kiharae*. With primers to two defensin-like genes from *T. aestivum* (AIA66989.1 and AIA66994.1), an amplification product encoding the putative defensin named Tk-AMP-D7 was obtained. Amplification with primers to *T. aestivum* Tad1 defensin gene (BAC10287.1) and two other defensins (AIA66993.1 and AIA66987.1) produced a single sequence named Tk-AMP-D8. Amplification with primers to *Ae. tauschii* defensin (XP_020164918.1) generated a defensin sequence named Tk-AMP-D9, which was 98% identical to the corresponding *Ae. tauschii* defensin and 100% identical to *T. urartu* defensin-like protein 1 sequence (EMS65298.1). Amplification with primers to two other defensins from *Ae. tauschii* (XP_020195731.1 and XP_020172230.1) produced two sequences named Tk-AMP-D10 and Tk-AMP-D11, respectively. All identified sequences, except for Tk-AMP-D9, were similar but not identical to the relevant *T. aestivum* and *Ae. tauschii* sequences and therefore represent novel defensin genes expressed in seedlings, which emerged from PME-treated *T. kiharae* seeds.

### 2.3. Assessment of PME Influence on Production Some Regulated Fusariotoxins

Since one of most disturbing consequences of wheat plant colonizing by Fusarium fungi is grain contamination with different mycotoxins, we examined if PME can serve as a potential tool to prevent or reduce the production of some of them and made sure that the extract did not stimulate this process. The effect on deoxynivalenol (DON), its monoacetylated derivatives (AcDON) and (zearalenone, ZER) production was evaluated on wheat gain treated with PME before artificial inoculation with toxigenic strains of *F. culmorum* (DON and AcDON producer) and *F. graminearum* (ZER producer) and germinated then under controlled condition. In addition, the level of DON production was determined in CL of *F. culmorum* grown in a submerged culture at the presence of PME.

No increase in the content of trichothecene mycotoxins DON and AcDON as well as a polyketide mycotoxin zearalenone was revealed when two FCR agents (*F. culmorum* and *F. graminearum*) developed on PME-treated seeds and their seedlings (Table 2). AcDON and ZER were produced by the toxigenic strains at the control level (as on water-treated seeds) independently of the PME concentrations used, while DON demonstrated a trend to the dose-rate dependence. A statistically significant decrease in DON accumulation was detected in inoculated wheat seedlings after applying the PME at a concentration of 0.5 mg/mL (Table 2) effective in laboratory assays and taken for field trails. It was also found no increase in DON production in vitro when *F. culmorum* were cultured on PME-containing media (Table 2).

### 2.4. Protection Wheat Plants under Field Conditions by Pre-Sowing Treatments of Naturally Infected Seeds with PME

To study the protective effect of PME under field conditions, grain samples of two spring wheat cultivars (Enita and Zlata) naturally infected with FCR and CRR were used. Each year before sowing, the grain intended for field trials were germinated in rolled up filter paper towels followed by mycological analyses of the grown seedlings that showed the presence of both *Fusarium* spp. and *B. sorokiniana* in all tested grain samples. *F. avenaceum*, *F. graminearum*, *F. culmorum*, *F. oxysporum*, *F. sporotrichioides* were revealed as FCR agents in Enita and Zlata seeds; *F. verticilloides* was isolated only from Enita grains in 2013. In all samples, *F. culmorum* was the predominant species. Content of seeds infected by *Fusarium* spp. were 40, 15, 20 (Enita) and 21% (Zlata) in 2013, 2014, 2015 and 2016, respectively. The incidence of *B. sorokiniana* on Enita seeds was 7, 5 and 9% in the samples of 2013, 2014, 2016, and reached to 15% in the sample used in 2015.

The treatment with PME did not influence the seed germination time in field and plant tillering capacity. Seedlings from treated and control seeds emerged simultaneously. The percent emergence on control plots ranged for the test years from 88.5 to 90.8, and averaged 90.6 ± 1.01, 89.0 ± 1.41, 88.9 ± 2.02 and 91.0 ± 2.01 on treated plots in 2013, 2014, 2015 and 2016, respectively. At the tillering stage, the tillering coefficient values of plants on control plots were 1.2–1.4 for Enita in 2013–2015, 1.6 for Zlata in 2016 and did not differ from that in plants grown from PME-treated seeds.

For all four years, seed incubation in PME solutions resulted in a significant reduction of FCR incidence and severity on spring wheat both at tillering (Z29) and early dough (Z83) growth stages (Table 3).

The disease development on seedling and plants was suppressed overall observation period, even if 40% of seeds in the sample (Enita, 2013) exposed to PME were contaminated with *Fusarium* spp. In addition, PME diminished field incidence of CRR on cv. Enita in 2013 and 2014 as well as on cv. Zlata in 2016 but diminishing in the incidence on Enita was insignificant in 2015 when the grain sample with 15% content of seeds infected by *B. sorokiniana* was used for treatment. In the last case, CRR mitigation was provided due to 3-fold (Z29) and 2,5-fold (Z83) reduction of disease severity index (DI%). Overall years, protection effectiveness against both diseases for both cultivars was higher at Z29 stage compared to Z83. The level of protective effect, which was calculated as explained below (see Materials and Methods, 4.11), for cv. Enita varied from 34% to 56% (FCR incidence) or from 36% to 46% (FCR severity) depending on the growth stage and test year, while cv. Zlata was protected with efficiency of 45–48% (both the incidence and the severity) independently on growth stage. The highest suppression of both diseases compared to control on cv. Enita occurred in 2013, when the incidence decreasing amounted up to 55.7 and 50.3% for FCR and CCR, respectively, while the maximal levels of severity mitigation were 46.3 (FCR) and 78.1% (CCR).

No significant difference in reduction of FCR and CCR development was found when wheat seeds were exposed to the extract sample that was stored frozen for six months and thawed before the treatment (Pme-20C in Table 3). At the same time, severity-reducing activity of this sample was saved after storage at −20 °C and freeze–thawing. Thus, pre-sowing immersion of Zlata seeds in Pme-20C resulted in not so high plant protection as in case of exposure to fresh-prepared PME but produced a statistically significant DI% diminishing compared to control (Table 3).

At the stem-elongation stage, PME application on Enita seeds did not promote development of productive stems in this cultivar but increased their number in Zlata plants at a statistical level (*p* = 0.05) compared to untreated plants (Table 4). The weight of 1000-grains as well as averaged yield per plot were responsive to both Enita and Zlata seed treatments. In our experiments, statistically significant enhancement of these parameters was recorded in 2013, 2014 and 2016. Consequently, the yield on plots with plants grown from the treated seeds exceeded the yield on the control plots by 7–9% in these years (Table 4). No yield component improvement and any economically important additional crop were obtained in 2015 (Table 4) when the lowest protection efficacy (see Table 3) was observed.

To assess the yield contamination by root rots, Enita and Zlata grains were sampled, in 2013 and 2016, respectively. Averaged samples of the grain harvested from PME-protected and control plants (800 kernels in each) were inspected using rolled-towel assay. These experiments revealed much less rotted seedlings emerged from kernels of plants grown from seeds treated with PME (21.8 ± 2.0% for Enita; 18.3 ± 0.7% for Zlata) compared to control (38.5 ± 1.5% for Enita; 28.8 ± 3.4% for Zlata). Thus, pre-sowing application of the extract on wheat seeds promoted grain sanitation and infestation control in the new yield.

## 3. Discussion

Along with hyperparasitism and competition, antibiosis is implemented as a powerful strategy for plant disease managing, which are based on natural mechanisms involved in microbe-microbe and plant-microbe interactions. As other microorganisms, endophytic and saprophytic fungi (including *Fusarium* strains) producing fungitoxic secondary metabolites, lytic enzymes, and elicitors of plant defense responses are used as biocontrol agents [5,38,39,40,41,42,43,44]. Their application for biocontrol is prospective because enable the suppression of several pathogens and is generally less damaging for environment than chemical pesticides. Fungal extracts containing these compounds, likewise other natural products extracted from plants or other sources, may be applied to combat plant diseases caused by pathogenic fungi [45,46,47,48] even if metabolic profile of the extracts is incompletely studied. Compared to commonly used living antagonists, preparations based on extracted fungal metabolites with protective activity would give additional potentialities for soil-borne disease managing. The majority of currently studied microbial agents are mesophiles that provide effective antibiosis over a relatively narrow range of environmental conditions. This is a main limitation to successful application of biological control in commercial agriculture against soil-borne pathogens in many countries including Russia. At the same time, the use of the remedies based on fungal metabolites would help to avoid the drawback of decreasing in biological activity of the microbial inoculants in soil and the rhizosphere of plants under highly varied external conditions. In our previous studies of FS-94 ability to induce the resistance against Fusaruim wilt, we showed that only seed treatment with viable germinating conidia of this non-patogenic strain protected tomato seedlings, while the conidia killed by autoclaving did not do this [33]. These data suggested the biocontrol effect found was very likely due to metabolites produced by the fungus. Our experiments on protection of wheat seedlings against six *Fusarium* species and two *B. sorokiniana* strains using seed treatment with PME confirmed this guess. The significant FRR and CRR mitigation, especially reduction of DI% values, demonstrated in field trials during four different growing seasons as well as after storage and freeze–thawing suggests protecting activity of PME should be stable under varying environmental conditions.

Noteworthy, managing of the diseases throughout the growing season was achieved in each year after single exposure of wheat seeds to PME. We have reasons to suppose, such a durable effectiveness of the pre-sowing treatments could be resulted from the enhanced level of plan defense responses, which activation was demonstrated both in vitro on cultured wheat cell and *in planta* on young seedlings. Thus, PME addition in cultures of *T. aestivum* and *T. kiharae* induced the ion exchange response of their cells, a well-known early non-specific defense reaction triggering a cascade of local molecular events and systemic biochemical signals and governing plant-pathogen interactions [49,50]. In addition, 16–18 hours incubation of cultured wheat cells with PME made them more tolerant to subsequent injury by *F. culmorum* that was the most prevalent causative agent of wheat root rots over all experimental years. Furthermore, the expression of genes encoding defensins, plant antimicrobial peptides (AMP) playing important roles against invading plant pathogens, was found in young wheat seedlings after the 3–day seed exposure to PME. Interestingly, our further investigation of the whole transcriptome in *T. kiharae* young seedlings exposed to a resistance-inducing fraction of PME, which was carried out using next generation sequencing technology (Odintsova at al., under review), showed that two of the defensin genes encoding Tk-AMP-D8 and Tk-AMP-D9 were up-regulated (fold change ≥ 2) in PME-treated and resistance-expressing seedlings. AMP are important components of the plant immune system [51,52]. Structurally diverse they are classified into several families [53], and defensins represent the most abundant AMP family being discovered in plants [54]. They belong to a distinct class of PR-proteins induced in plants upon pathogen attack [55]. Transgenic plants overexpressing defensin genes become more resistant to biotic stress caused by pathogens and pests, thus highlighting the significant role of defensins in the defensive arsenal of plants [54]. Earlier, in seeds of the highly resistant to pathogens species *T. kiharae*, we discovered and sequenced as many as 13 defensins grouped into three subfamilies according to their N-terminal sequences, the most abundant of which were named Tk-AMP-D defensins [56,57]. In the current work, we used these findings to assess one of possible mechanisms of plant-mediated mode of PME action, which could underlie the durable control of FCR and CRR on wheat plants and showed that seed treatment with the tested extract elicited expression of several defensin-like genes in roots and leaves of young seedlings. Their products are highly similar to Tk-AMP-D and Ec-AMP-D1, defensins earlier described in seeds of wheat and barnyard grass (a weed cereal), respectively, which were shown to be active against several phytopathogenic fungi, including *Fusarium* spp. and *H. sativum* (syn. *B. sorokiniana*) at micromolar concentrations [56,58]. In addition, data presented here confirm results of other authors showed the role defensins in protection of wheat and other plants against root diseases that are inefficiently controlled by fungicides [31,32]. Thus, we can expect that gene expression of these defensive peptides in seedlings grown from PME-treated seeds might also contribute to wheat protection against root rot causative agents under field conditions.

The study of microbial metabolome ability of triggering or priming defense responses is of special interests for plant protection because their components do not target directly the pathogen [59] and hence, they do not promote establishing the resistance in pathogens and pests, which is a serious problem accompanying application of chemical and even biological pesticides [60,61]. However, some induced defense responses, e.g. generation of reactive oxygen species (ROS) and the synthesis of certain plant secondary metabolites, are factors of biotical stress for invading fungi [62,63]. As a result of the oxidative stress, various pathogenic fungi activate the biosynthesis of polyketide mycotoxins [64]. Besides, in wheat pathogenic *Fusarium* species, these responses supposedly save as signals to increase the biosynthesis of trichothecene mycotoxins [65]. The ion exchange response, which was induced in cultured wheat cell by PME, have been reported may occur concurrently with ROS generation [66], thus promoting mycotoxigenesis in FCR agents. In this context, before testing the FS-94 extract as an agent managing wheat root rots in the field, we carried out laboratory tests to confirm that wheat seed treatment with PME did not stimulate FCR agents to boost production of some widespread fusariotoxins and showed PME did not potentiate the production at least three of hazardous mycotoxins strongly regulated in food and feeds.

Summing up of all presented results points to the tested FS-94 extract may be considered as a promising tool to manage wheat diseases caused by *Fusarium* spp. and *B. sorokiniana*. However, here, we reported only laboratory evidence of PME plant-mediated mode of action and activation of wheat defense responses. Additional research to determine if these mechanisms are involved in the protective effect observed under field conditions is needed. For optimal implementation of PME potentialities in biocontrol of root rots, it is necessary to study its composition in more detail and identify components responsible for plant resistance induction. Also, quantification of not only DON, AcDON, zearalenon but also other mycotoxins in harvested grain should be included in further investigations.

## 4. Materials and Methods

### 4.1. Fungal Cultures

*Fusarium sambucinum* FS-94 as well as pathogenic Fusarium strains (*F. culmorum* OR-02-37, *F. avenaceum* Br-04-60, *F. graminearum* FG-30, *F. sporotrichioides* KRT-12-1, *F. oxysporum* KF-1713-4, *F. gibbosum* KU-8-4, *Bipolaris sorokiniana* Ir-01-38 and KrD-81) were obtained from the State Collection of Plant Pathogenic Microorganisms at All-Russian Research Institute of Phytopathology.

### 4.2. Purification of Mycelium Extract and Preparation of CL Filtrates

For experiments requiring mycelium extracts and CL filtrates, 10 mL of a 24-hour starter culture of FS-94 grown on liquid Raistrick's medium were added in 750 mL Erlenmeyer flasks containing 100 mL of liquid Czapek’s medium (pH 6.2) supplemented with molasses (60 g/L) and peptone (0.8 g/L). The flasks were incubated on an orbital shaker at 220 rpm and 25–26 °C in the darkness for 2 days.

Fungal mycelium and CL were separated by vacuum filtration through sterile Miracloth. The CL was passed through a GF/A Whatman filter [36], and the resulting filtrate was freeze-dried.

The mycelium was thoroughly washed with a fivefold volume of freshly prepared distilled water followed by collection and removal the water excess by squashing between several layers of sterile filter paper. The pressed mycelium was ground under liquid nitrogen, extracted and purified by gel filtration on Sephadex G-50 as described previously [44] to obtain a combined fraction that eluted by 0.05 M Na/K phosphate buffer in a volume of Blue Dextran (2000 kDa). Samples of gel-filtrated extract were further purified from the buffer salts and unutilized components of the nutrient medium by ultrafiltration in Stirred Ultrafiltration Cell, (Amicon) with a 3 kDa-cut-off membrane (Ultracel®- NMWCO 3K). The over-membrane concentrates (retentates) were washed several times with dW, collected and freeze-dried.

Just prior to laboratory assays, lyophilized preparations of the PME and the CL filtrates were dissolved in distilled water to obtain solutions with final concentrations ranged in different experiments from 0.05 to 1 mg/mL, which were sterilized by passing through a 0.22 μm Millipore membrane filter.

Freeze-dried PME preparations for field trials were dissolved on the treatment day except one PME portion applied for immersion of Zlata seeds (referred above as Pme-20C), which was obtained six months before the experiment, dissolved, stored at −20 °C and was melt an hour prior to seed treatment.

### 4.3. Seed Treatment

Seeds of two spring wheat cultivars (Enita and Zlata) were surface-disinfested by immersion in 0.5% KMnO_4_ for 10 min, and then triply rinsed with sterile distilled water (sdW). The seeds were dried a little by blotting them with sterile filter paper, soaked in PME or sdW (500 seeds/50 mL) and incubated on a laboratory shaker for 3h at gentle agitation followed by the overnight incubation at 20–22 °C without an agitation.

For field experiments, treatments of seeds naturally infected by the pathogens were carried out by the same way except the grains were not surface-disinfested before soaking in PME solutions.

### 4.4. Artificial Inoculation of Seeds

To prepare pathogenic inoculums for laboratory experiments, strains of above listed pathogens (see Materials and Methods, Section 4.1) were grown in 9 cm Petri dishes on potato dextrose agar (PDA) at 25 °C in the dark for 7–10 days. About 20 mL of sterile distilled water were pipetted in a dish, and conidia were gently scraped from the colony surface. The suspension was filtered through a sterile cotton wool to remove mycelium debris and centrifuged at 3000 g for 10 min. The suspensions were diluted to concentrations 1–1.5 × 10^6^ conidia/mL and used in a rolled-towel assay to inoculate Enita seeds by application of the 100 µL aliquot on each kernel.

### 4.5. Rolled-Towel Assay for Estimation of PME Protection Effect

Inoculated seeds were placed 1 cm distance one another along a central horizontal line of a sterilized filter paper towel size of 10 × 50 cm (four towels per treatment, 50 kernels in one towel). Seeds were covered by a tracing-paper film (8 × 50 cm). Another paper towel was placed over the film. Both towels and film were slightly moistened prior to usage. Towels with seeds were rolled up and set vertically in 250 mL glass beakers with distilled water to cover lower part of towels but not seeds. The rolls with non-inoculated seeds were prepared to ensure that other pathogens were not present. The beakers were placed under a transparent plastic bucket, and wheat seedlings were grown in a climatic chamber at 22 °C (day), 18 °C (night) and 60% relative humidity for 10–14 days.

To determine the incidence of the diseases, the number of symptomatic seedlings was counted, and their percentage to the total count of seedlings was calculated. To evaluate the DI%, the following five-point scale and the formula were used:
$\mathrm{DI},\%=\frac{\Sigma \left(d\times n_{1}\right)}{4n}\times 100$0 - no disease symptoms 1- weak symptoms (brown streaks or spots) on roots and above2 - are manifest, but medium symptoms3 - strong extensive symptoms 4 - crown and root decaying or plant death

n_1_ – the number of seedlings with the same index; d - index according to the rating scale (from 0 to 4);
n – plant number per treatment.

The rolled-towel assay was also applied to investigate pre-sowing incidence of FCR and CCR in naturally infected seeds used in small-plot experiments as well as post-harvest root rot incidence in the grain sampled after these trials.

### 4.6. Mycological Analysis of Seeds

To confirm the presence of FCR and CCR agents and determine what Fusarium fungi were presented in naturally infected wheat grain intended for field trials, the averaged samples of 100 randomly selected seeds were disinfested and washed as described above. The disinfested seeds were transferred to Petri dishes and planted on Selective Fusarium Agar (SFA) [67] or PDA [68] to reveal *Fusarium* species or *B. sorokiniana,* respectively. The dishes with seeds were incubated at 25 ℃ in the dark for 5 days. Fungal colonies growing on SFA were sub-cultured and *Fusarium* fungi were identified based on the micro- and macro-morphological features such as characteristics of conidia, chlamydospores, mycelium, and colony appearance, color, and shape [69]. Conidia of *B. sorokiniana* washed-out from the PDA surface were identified directly under the microscope.

### 4.7. In Vitro Testing the Growth and Conidium Germination of Root Rot Agents Exposed to PME

To ascertain if the tested FS-94 extracts possesses an antifungal activity against FCR and CCR agents, conidia of the pathogenic *Fusarium* strains and *B. sorokiniana* were suspended in PME with concentration 0.5 mg/mL, followed by diluted the initial suspension with the same extract to a final concentration of 10^6^ conidia/mL, and germinated in a thin agar layer as described previously [70]. The average percentage germination relative to control (conidia that germinated in sterile distilled water) was calculated for 300 conidia of each pathogen. PME influence on radial growth of the FCR and CCR agents were examined by culturing the fungi on 1.5% Czapek’s containing 0.5 mg/mL of PME that was added at stirring in molted Czapek agar at 40 °C. After solidification, the media were inoculated by the pathogens inspected daily for 10 days to found a growth inhibition by comparison of the pathogenic colonies to control colonies on PME-free Czapek agar.

### 4.8. Studying the PME Effects on Cultured Wheat Cells

Wheat seeds were sterilized by immersing successively in 95% ethanol, 1% AgNO_3_ and 10% H_2_O_2_ for 10, 25 and 10 min, respectively. Before and upon incubation in 10% H_2_O_2_, seeds were washed six times with sterile distilled water, slightly dried and placed in Petri dishes (6 kernels in a dish) containing Murashigue and Skoog (MS) agar supplemented with 2,4-D (15 mg/L) and α-naphtylaneacetic acid (1 mg/L) for callus induction [71]. The dishes were incubated in a growth culture room at 25 °C and 16h light period for 10–15 days. The calli formed were separated from seeds by a sterile metallic scalpel, transferred on MS agar amended with 2 mg/L of 2,4-D (MSD), and sub-cultured for 1–1.3 months.

If subsequent experiments involved co-culturing wheat cells with *F. culmorum* conidia, about 0.5 g fresh weight of granular callus were transferred into 750 mL shaker flasks containing 100 mL of liquid MSD medium and incubated them on an orbital shaker New Brunswick E-25R at 200 rpm and 26 °C in the dark for 2 or 4 weeks (in the last case, 25 mL of the fresh medium were added after 2 weeks). Wheat cells were precipitated by centrifugation at 2000 g for 10 min in sterile tubes and suspended in sterilized PME (0.5 mg/mL) to produce a final concentration of 35,000–50,000 cells/mL. After overnight incubation, the cells were re-centrifuged and transferred to a fresh portion of MSD. The suspension was combined with conidia of *F. culmorum* (3 conidia per 1000 cells) and co-incubated with the pathogen at 26 °C and 150 rpm for 24, 48 or 72 hours. A portion of the wheat cell suspension supplemented with fungal conidia killed by autoclaving saved as a control. Upon co-incubation, the concentration of unlyzed cells was determined using a Fuchs-Rosenthal Counting Chamber.

To assay PME-induced extracellular alkalization in suspension cultures of *T. aestivum* L. (cv. Enita) and *T. kiharae* Dorof. et Migusch. cells, they were cultivated on an orbital shaker in the dark at 140 rpm and 25 °C in 50 mL flacks with 25 mL of liquid MS containing sucrose (30 gL/L), myoinositol and asparagine (100 mg of each), 2,4-D (2,5 mg/L), glycine, thiamine, and pyridoxine (2.0 1.0 and 0.5 mg/L, respectively). Sub-culturing was carried out every 14 days by the addition of 5 mL of the suspension to 20 mL of fresh medium. The tested extract of FS-94 mycelium was concentrated by ultrafiltration and pipetted in a minimal volume into 8-mL portions of 6–8-day-old cell cultures to PME concentrations 0.1 mg per mL of the suspension. A reversible response of wheat cells to PME was analyzed according to the method elaborated by Felix et al. [72].

### 4.9. Gene Expression Assay of Defensins in Germinated Wheat Seeds

*T. kiharae* seeds were surface sterilized in 1% KMnO_4_ for 15–20 min, then washed thoroughly in sterile distilled water and placed on sterile paper filters in Petri dishes (35–50 seeds were dish). Surface-disinfested seeds thoroughly washed in sdW were placed on sterile paper filters in Petri dishes (35–50 seeds in each one). Two milliliters of sterile distilled water were added to each Petri dish, and seeds were further incubated at 20–22°C for 6 h. Imbibed seeds were treated with PME (50 µL per seed) and grown for 3 days at 20–22°C, the first day in the dark, and then two days under long-day conditions (16 h light/8h dark), radicles and embryonic shoots with first leaves were harvested, immediately frozen in liquid nitrogen and stored at −80°C for isolation of total RNA using the RNAwiz reagent (Ambion, Canada) according to the manufacturer’s protocol. RNA preparations were treated with DNase I (Fermentas, Lithuania) and reverse transcribed using Revert Aid First Strand cDNA Synthesis Kit (Fermentas, Lithuania) and oligo(dT)_18_ primer. The amplified cDNAs coding for specific defensin-like genes were synthesized using high-fidelity Tersus DNA polymerase (Evrogen, Russia), and gene-specific primers (Table 5) were designed using the NCBI/Primer-Blast program.

PCR conditions were as follows: initial denaturation step at 94 for 2 min followed by 36 cycles of denaturation at 94 °C for 30 s, primer annealing at 59 °C–60 °C for 30 s, and primer extension at 72 °C for 30 s, with the final extension of 5 min at 72 °C. β-Actin gene (AB181991.1) was used for normalization of cDNA samples (Act-forward (5ʹ-TTTGGCCTCTCTTAGCACTTTC-3ʹ) and Act-reverse (5ʹ-AGGAAAAGCTGAACCGAGAC-3ʹ)), PCR fragment length is 237 bp. PCR products were separated in 2% agarose gels. Isolation of DNA fragments was carried out with the Cleanup Mini kit (Evrogen, Russia). PCR fragments were cloned into the pAL2-T vector (Evrogen), and at least five clones were sequenced on an ABI PRISM 3730 sequencer (Applied Biosystems, USA).

### 4.10. Mycotoxin Quantification After Artificial Seed Inoculation with Toxigenic Fusarium Strains and in Submeged Culture of F. Culmorum.

Surface-disinfected Enita seeds pre-soaked in aqueous PME solutions (0.2, 0.5, 1.0 mg/mL) or in in sterile distilled water (control) were artificially inoculated by conidia of toxigenic *F. culmorum* or *F. graminearum* strains and germinated in paper rolls as described above (see Section 4.4 and Section 4.5) for 7 days. Infected kernels with roots were dried at 42 °C in a thermostat and ground into flour in a laboratory mill. To study the influence of PME on in vitro DON production by *F. culmorum*, the fungus was gown in a submerged culture at 220 rpm and 25 °C for 7 days on toxinogenesis-promoting liquid Myro medium [73,74] supplemented with the extract to the final concentrations of 0.5 or 1.0 mg/mL. The fungus culture from PME-free medium obtained under the same conditions served as a control. The ground samples or the fungal broth cultures were extracted several times with five (*w*/*v*) or three (*v*/*v*) volumes of the extraction solvent mixture (acetonitrile-water-methanol in a ratio of 20:4:1, *v*/*v*) in Erlenmeyer flasks at continuous vigorous shaking for 30 min (ground grain) or in a separating funnel (fungal broth) and filtered through Whatman™ paper filters. The obtained filtrates were purified either by absorption chromatography or by redistribute extraction with immiscible solvents. Briefly, to isolate DON and AcDON, a portion of the filtrate was applied on a self-designed activated charcoal-alumina column (1:1) and eluted with the mixture acetonitrile-water (5:1, *v*/*v*). To recover ZER, another portion of the filtrate was evaporated to aqueous phase that was adjusted with a saturated solution NaCl to 20 mL and extracted three times by the equal volume of water-saturated hexane in a separating funnel. After separation, the aqueous phase was triply extracted with dichloromethane and passed through a layer of anhydrous Na_2_SO_4_. Upon completion of clean-up procedures, seed extracts were evaporated to dryness on a rotary evaporator. The residues were dissolved in minimal volumes of acetonitrile-methanol (1:1), filtered through a 0.45-μm pore size membrane and analyzed, using Waters1525 Breeze HPLC system equipped with a Waters 2487 UV detector (Waters Corp, USA). Samples (10 μL) were applied on the Symmetry C18 (5µm, 150 × 4.6 mm) temperature-controlled (27 °C) column (Waters Corp., USA), eluted with acetonitrile-methanol-water (1:1:7-trichothecenes; 1:1:0.75-ZER) at rate 0.8 mL/min and detected at 254 nm. The mycotoxin concentrations (μg/g) were calculated using a calibration curve based on commercial single-component preparations of DON, DON derivates (3- and 15-AcDON) and ZER (Sigma-Aldrich Corp.). They were added in control extracts from non-inoculated seeds (3- and 15-AcDON were used as a mixture 1:1) or in the control fungal broth culture (DON) and were also used as external standards. All samples were analyzed in triplicate. The minimal detectible concentration was 0.5 (DON, AcDON) and 0.2 (ZER) μg/g, the recovery was 80%, 75% and 73% for DON, ZER and AcDON, respectively.

### 4.11. Small-Plot Field Experiment Design

To test the effect of PME on FCR and CRR development under field conditions, small-plot experiments on pre-sowing seed treatment of two spring wheat cultivars (Enita and Zlata) regionalized for Non-Chernozem zone of Russia were carried out in Moscow region during cropping seasons of 2013–2016 (cv. Enita) and 2016 (cv. Zlata). A different field and different seed batches were used in each year, but soil type (sod-podzolic, medium loamy, weakly acidic with the cloddy structure and dark-gray arable layer) and the preceding crop (potato) were the same for all test years. During plowing, the soil was fertilized with N-90, P-120, K-100 (kg/ha) as recommended for Moscow region. In each experiment, a randomized complete block design with 1m^2^-plots in four replications per a treatment was used. Plots were seeded at a rate of 500–550 seeds/plot on May 24, May 23, May 20, and May 19, in 2013, 2014, 2015 and 2016, respectively. No fungicides or insecticides were applied on plants, manual weeding was done at the growth stages Z25-30 [75]. Seedlings was counted on each plot in one-two weeks after sowing when first leaves emerged (Z11), and percent emergence was calculated relative to seeds sown per plot. The ratio of shoot number to plant number was determined at Z29 (main shoot and nine or more tillers) and referred to as the tillering coefficient.

To evaluate incidence and severity of root rots, 30–50 seedlings at the end of the tillering stage (Z29) were randomly collected from a 50 cm length of each rows in each plot (i.e. from randomly selected 2 m length per plot). Wheat plants for the second examination were similarly sampled at the early dough (wax ripeness) stage (Z83). Plants were carefully removed from soil and roots were thoroughly washed with tap water before assessment. FCR and CRR development was evaluated by percent symptomatic plants (incidence) and DI% value using above five-point rating scale (see Section 4.5). The level of protective effect expressed as percentage was calculated according to formula Xc-Xt/Xc × 100, where Xc and Xt meant average incidence or DI% in control or in treatments, respectively.

At the end of vegetative seasons, all plants on plots were harvested manually and air-dried to 14 ± 0.2% kernel moisture content. The number of productive stems, total grain weight from one plot, and weight of 1000 kernels were averaged. Then, average weight of a spike and average yield were calculated.

### 4.12. Statistical Analysis

Quantitative data of the experiments were statistically analyzed with Microsoft software STATISTICA 6.0 (StatSoft Inc.). Means of different treatments, standard errors or standard deviations, and significant differences (*p ≤* 0.05) of means between treatments and controls were determined using a *t*-test for independent variables.

## Figures and Tables

**Table 1:** Difference in the survival ability of pre-treated and untreated wheat cells during cultivation in a suspension culture inoculated with *Fusarium culmorum*.

Treatment	Concentration of Unlyzed Cells **, 10^3^/mL		
	24 h	48 h	72 h
Untreated cells *	58.3 ± 3.69	58.4 ± 6.03	55.0 ± 6.23
Cells + PME	60.9 ± 4.01	56.4 ± 3.94	54.7 ± 4.52
Cells + *F. culmorum*	28.1 ± 1.53	10.7 ± 1.34	5.2 ± 1.62
Cells + PME + *F. culmorum*	40.4 ± 2.56	33.3 ± 2.80	17.0 ± 1.39

* Initial concentration of suspension averaged 62.1 × 10^3^ wheat cells/ml. ** Results represent means values ± SD of two experiments, three replications per treatment in each.

**Table 2:** Contents of mycotoxins in wheat seeds non-treated or treated with purified extracts from FS-94 mycelia (PME) and prior to inoculation by trichothecene- or zearalenone-producing *Fusarium* strains, and production of deoxynivalenol by *Fusarium culmorum* cultivated on PME-containing media.

PME, mg/mL	Mycotoxins, µg/g			
	Grain			Submerged Culture
	*F. culmorum*		*F. graminearum*	*F. culmorum*
	DON	AcDON	ZER	DON
0.2	78.54 ^a^	10.63 ^c^	0.78 ^d^	not used
0.5	74.69 ^b^	12.88 ^c^	0.86 ^d^	0.376 ^e^
1.0	61.14 ^b^	10.55 ^c^	0.67 ^d^	0.374 ^e^
0 *	89.96 ^a^	14.99 ^c^	0.94 ^d^	0.432 ^e^

DON- deoxynivalenol, AcDON-monoacetylated derivatives of DON, ZER-zearalenone. * Seeds treated with sterile distilled water. Differences between values denoted with the same small case letters (a, b, c, d ,e) are insignificant (*t*-test, *p* = 0.05).

**Table 3:** Effect of pre-sowing seed treatment with PME on FCR and CCR development on spring wheat plants.

Test Year, Cultivar	Seed Treatment	Development of Root Rots Caused by							
		*Fusarium* spp.				*B. sorokiniana*			
		Incidence		DI, %		Incidence		DI, %	
		Z29	Z83	Z29	Z83	Z29	Z83	Z29	Z83
2013, cv. Enita	Control *	53.7	72.3	17.7	22.6	12.7	15.9	6.4	6.7
	PME	23.8	43.3	9.5	13.6	6.3	9.1	1.4	2.8
	*p=*	*0.03*	*0.002*	*0.04*	*0.01*	*0.02*	*0.05*	*0.003*	*0.01*
2014, cv. Enita	Control *	52.6	67.9	18.5	20.6	14.3	17.6	4.6	8.8
	PME	31.5	42.9	10.4	12.5	7.7	11.4	1.3	4.2
	*p=*	*0.04*	*0.02*	*0.05*	*0.05*	*0.01*	*0.05*	*0.01*	*0.02*
2015, cv. Enita	Control *	44.0	55.3	12.1	18.2	23.4	25.5	3.9	6.8
	PME	22.6	36.5	7.5	11.6	19.4	22.7	1.3	2.7
	*p=*	*0.05*	*0.004*	*0.05*	*0.05*	*0.07*	*0.08*	*0.04*	*0.03*
2016, cv. Zlata	Control *	46.7	54.6	15.7	17.9	6.9	10.9	3.0	4.3
	PME	25.8	29.6	8.3	9.7	4.1	5.3	1.2	1.8
	*p=*	*0.04*	*0.02*	*0.004*	*0.05*	*0.05*	*0.04*	*0.003*	*0.002*
	Pme-20C **	39.6	48.7	9.9	11.5	5.0	9.4	2.1	3.1
	*p=*	*0.06*	*0.08*	*0.03*	*0.05*	*0.09*	*0.09*	*0.04*	*0.05*

* Control seeds were treated with distilled water. ** Pme-20C is the dissolved sample of PME that has been frozen and stored at -20 °C for six months before the seed treatment. Z29 and Z83 are growth stages of wheat plants, which indicated according to Zadoks’ scale DI, %: disease severity index (see Materials and Methods, 4.5). Significant differences (*p* ≤ 0.05) of means between treatments and controls were determined using a t-test for independent variables. *P* values for each treatment at each of two growth stages in each year are indicated in italics.

**Table 4:** Effect of pre-sowing seed treatment with PME on yield components of two spring wheat cultivars.

Test Year	Seed Treatment	Yield Parameters *			
		Number of Productive Stems Per Plot	Weight of 1000 Kernels, g	Total Grain Weight from One Plot, g	Yield Increase, % of Control **
	cv. Enita				
2013	PME	490 ^a^	37.3 ^a^	540.1 ^a^	8.4
	Untreated seeds	483 ^a^	32.5 ^b^	498.4 ^b^	
2014	PME	523 ^a^	36.8 ^a^	533.8 ^a^	6.0
	Untreated seeds	498 ^a^	34.4 ^b^	486.2 ^b^	
2015	PME	475 ^a^	32.2 ^b^	501.9 ^b^	2.0 ***
	Untreated seeds	467 ^a^	30.6 ^b^	492.2 ^b^	
	cv. Zlata				
2016	PME	541 ^b^	37.2 ^a^	581.2 ^c^	7.3
	Untreated seeds	464 ^a^	33.8 ^b^	541.5 ^a^	

* Means for four randomized plots for each treatment in each year. Distinctions between means marked by the same letters (a, b, c) within a column for each parameter are insignificant at *p* ≤ 0.05. ** The grain yield on control plots in corresponding test year. *** The distinction from control is insignificant (*p* > 0.05).

**Table 5:** Sequences of primers used for isolation of *Triticum kiharae* defensin genes.

#	Peptide Accession Number	mRNA Accession Number and Annotation	Primer Sequences (5′→3′); Annealing Temperature (T),^0^C; Fragment Length (L), bp	*T. kiharae* defensin	Sequence similarity, (%)
1	AIA66989.1	KJ551519.1 T. aestivum, defensin (PDF4)	FOR AGCTGAGCAGATCGATGGCG REV GGCTAGCAGGCCCTCTTGCA T=60 L=265	Tk−AMP−D7	96%
2	AIA66994.1	KJ551524.1 T. aestivum, defensin (PDF9)			90%
3	BAC10287.1	AB089942.1 T. aestivum, defensin (Tad1)	FOR GTGAAGCGAGCAAGCAGAGAGA REV TAGGGACGAACAGATCTAA T=60 L=360	Tk−AMP−D8	94%
4	AIA66993.1	KJ551523.1 T. aestivum, defensin (PDF8)			98%
5	AIA66987.1	KJ551517.1 T. aestivum, defensin (PDF2)			94%
6	XP_020164918.1	XM_020309329.1 Ae. Tauschii, defensin Ec-AMP-D2-like (LOC109750363)	FOR GCTGCTCACACACAACACAC REV CAGAAAGGCCACCCGAAAGA T=59 L=372	Tk−AMP−D9	98%
7	XP_020195731.1	XM_020340142.1 Ae. Tauschii, AMP-D1.2-like (LOC109781554)	FOR GCTAGCTTTACACACAGCCC REV ACCGTAGCTAGCATCGGACC T=59 L=330	Tk−AMP−10	96%
8	XP_020172230.1	XM_020316641.1 Ae. Tauschii, defensin Tk-AMP-D2-like (LOC109757808)	FOR CAAGCAGAGAGATGGCGTCC REV CGGATGCATGGAGATGAACCA T=59 L=402	Tk−AMP−11	94%
9	AIA66999.1	KJ551529.1 T. aestivum, defensin (PDF14)	FOR AGAGAGCAAGTGCAGAAGAGA REV AAGCTCAGCAGTCCCGCTCGCA T=69 L=281	-	-
10	XP_020172235.1	XM_020316646.1 Ae. Tauschii, defensin Tk-AMP-D2 (LOC109757811)		-	-
